# Environmental ranges discriminating between macrophytes groups in European rivers

**DOI:** 10.1371/journal.pone.0269744

**Published:** 2022-06-14

**Authors:** Willem Kaijser, Sebastian Birk, Daniel Hering

**Affiliations:** 1 Faculty of Biology, University of Duisburg-Essen, Aquatic Ecology, Universitätsstraße 5, Essen, Germany; 2 Centre for Water and Environmental Research, University Duisburg-Essen, Universitätsstraße 5, Essen, Germany; Southeastern Louisiana University, UNITED STATES

## Abstract

Riverine macrophytes form distinct species groups. Their occurrence is determined by environmental gradients, e.g. in terms of physico-chemistry and hydromorphology. However, the ranges of environmental variables discriminating between species groups (“discriminatory ranges”) have rarely been quantified and mainly been based on expert judgement, thus limiting options for predicting and assessing ecosystem characteristics. We used a pan-European dataset of riverine macrophyte surveys obtained from 22 countries including data on total phosphorus, nitrate, alkalinity, flow velocity, depth, width and substrate type. Four macrophyte species groups were identified by cluster analysis based on species’ co-occurrences. These comprised Group 1) mosses, such as *Amblystegium fluviatile* and *Fontinalis antipyretica*, Group 2) shorter and pioneer species such as *Callitriche* spp., Group 3) emergent and floating species such as *Sagittaria sagittifolia* and *Lemna* spp., and Group 4) eutraphent species such as *Myriophyllum spicatum* and *Stuckenia pectinata*. With Random Forest models, the ranges of environmental variables discriminating between these groups were estimated as follows: 100–150 μg L^-1^ total phosphorus, 0.5–20 mg L^-1^ nitrate, 1–2 meq L^-1^ alkalinity, 0.05–0.70 m s^-1^ flow velocity, 0.3–1.0 m depth and 20–80 m width. Mosses were strongly related to coarse substrate, while vascular plants were related to finer sediment. The four macrophyte groups and the discriminatory ranges of environmental variables fit well with those described in literature, but have now for the first time been quantitatively approximated with a large dataset, suggesting generalizable patterns applicable at regional and local scales.

## 1. Introduction

Riverine macrophyte species occur under a wide range of chemical and hydromorphological conditions and can be classified into groups according to their ecological preferences resulting in distinct distribution patterns [1–3]. These distribution patterns are useful, for instance, to indicate environmental conditions relevant for target setting in ecological conservation and restoration. There is, however, limited quantitative empirical information on the environmental conditions where species groups differentiate along multiple gradients.

Chemical variables have received most attention in identifying potential discriminative ranges of macrophyte species, with nutrient concentration being studied most frequently. The input of nutrients increases algae growth, restricting light and promoting faster-growing, canopy-forming macrophytes which can outcompete smaller species [4,5]. Alkalinity, a proxy for dissolved inorganic carbon (DIC), is another important driver of macrophyte [6,7]. Thus, nutrients and DIC are major determinants for macrophyte occurrence.

Hydromorphological characteristics including flow velocity, depth, width and substrate type also strongly affect macrophyte occurrence [8–10]. Streamlined, small or deep-rooting macrophyte species are able to tolerate higher and turbulent flow in upstream shallow sections [11–13]. Especially mosses are able to tolerate higher flow velocities and grow on coarse and stable substrate while superficially rooting or free-floating species with large leaf areas prefer downstream sections, which are deeper, wider and characterised by lower flow velocities and finer sediment [2,10]. Therefore, habitat-related gradients pose an additional layer of complexity for understanding the composition of macrophyte groups.

Along all these different environmental gradients, we expect the occurrence of different macrophyte groups being discriminated by specific ranges of selected environmental gradients. Such “discriminatory ranges” denote gradient regions which separate between the occurrence of different macrophyte groups. “Groups” are defined as macrophyte species that often co-occur under similar conditions.

Currently, discriminative ranges are based on expert judgment or derived from small-scale experiments, rather providing descriptive accounts scattered across the scientific literature [14–16]–the question of discriminatory ranges is addressed with little empirically evidence. But what is the meaning of environmental descriptions such as ‘shallow’ or ‘deep’, ‘high nutrients’ or ‘low nutrients’ with regard to the occurrence of distinct groups of macrophyte species? Is it possible to substantiate these qualitative expressions with quantitative values?

In this article, we explore a large dataset spanning 22 countries in Europe, which covers large environmental gradients linked to the occurrence of river macrophytes. This allows us to address two research questions:

1. Which groups of co-occurring macrophyte species can be observed across Europe’s rivers?
2. What are the discriminative ranges of these macrophyte groups along gradients of nutrients, alkalinity, flow velocity, depth, and width at continental scale?

## 2. Material and methods

### 2.1 Data basis

#### Biotic data

**Fig 1:**
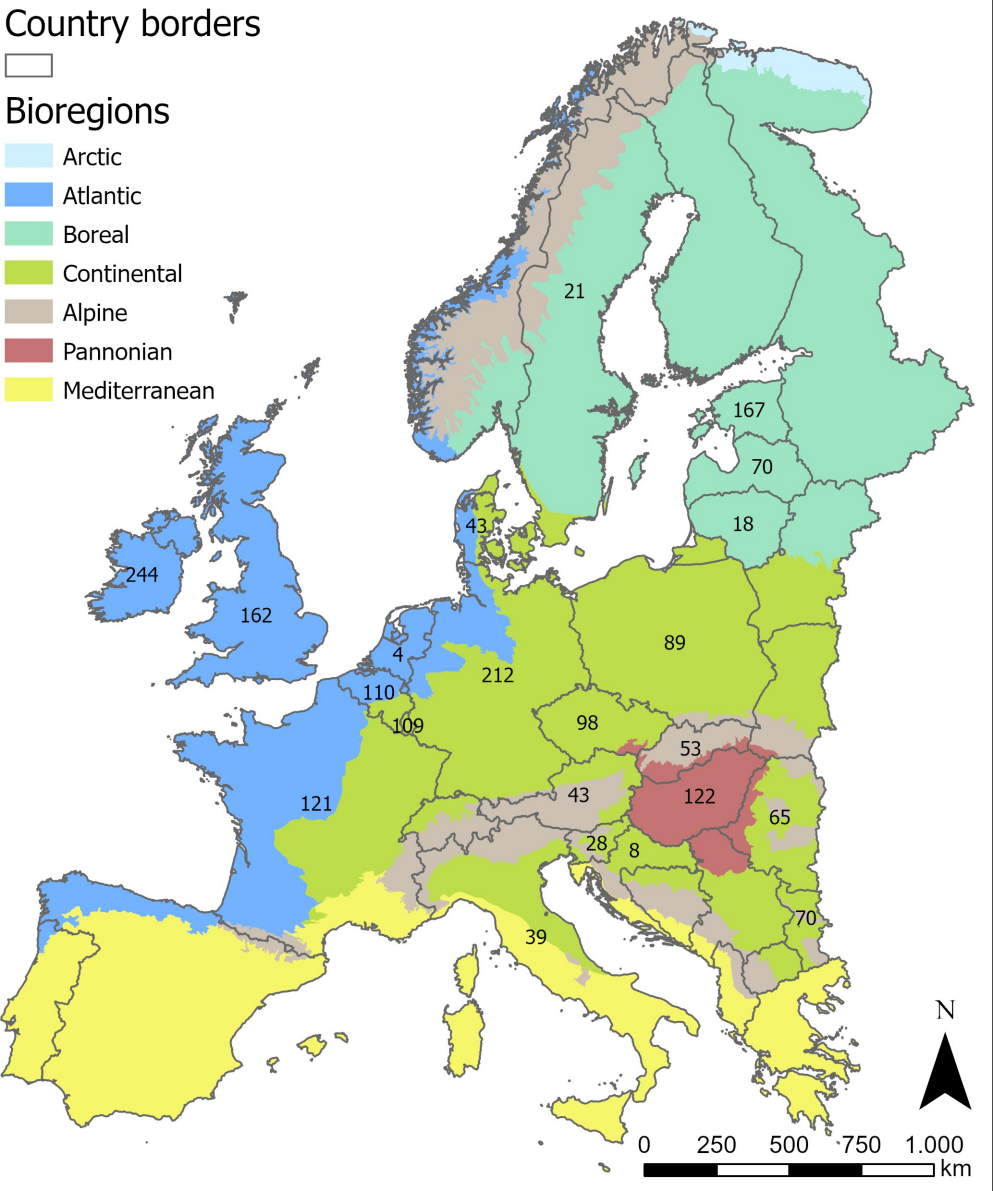
Map of Europe with bioregions and sample size per country.

**Fig 2:**
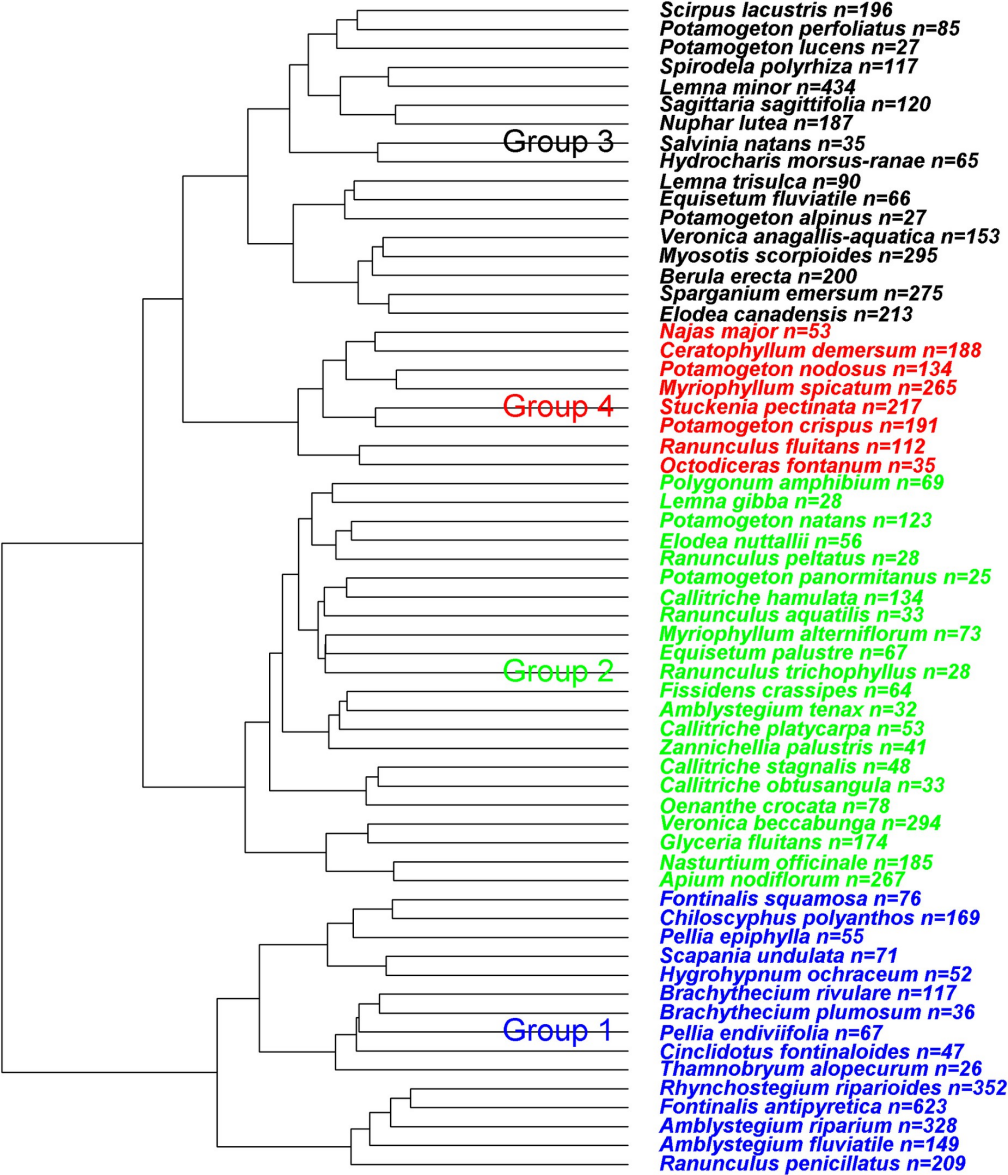
Dendrogram with four groups used in the RF models. n = number of species’ occurrences in the group.

We have used a dataset collated from national monitoring programmes to assess the ecological river status [17, see S1 Table for the respective data providers]. These data were initially used to compare the national classifications of ecological river status and covered 22 countries including all European bioregions except for the Boreal and Mediterranean region (both regions not part of this comparison, Fig 1). The rivers included in the dataset comprise a range of different types from small upland brooks, to medium-sized rivers and large lowland rivers (e.g., Danube River). All national protocols for the macrophyte surveys complied with the European Standard CEN-EN 14184 [18]. In short, riverine macrophytes were monitored along a river reach of 100 m during the growing season (June to September) by wading, diving or boating, using rakes or grapnels. No multiple samples from a single site were used (no duplicates). We only included macrophyte species with a high water-affinity [’aquaticity levels’ 1 and 2 according to [19,20] and excluded species recorded at less than 25 sites. This number was derived from initial data screening, chosen to prevent the grouping of species in non-representative clusters due to minimal sample size. This resulted in rarely occurring species being excluded from the analysis (e.g., *Chara globularis* with n = 3, *Potamogeton polygonifolius* with n = 15, *Ranunculus baudotii* with n = 1 or *Utricularia vulgaris* with n = 3). The dataset used in our analysis covered 1,896 unique sampling sites surveyed between 1994 and 2011 including 62 species and 8,090 species records (see Fig 2).

#### Environmental data

The associated environmental data covered hydromorphological as well as chemical surface water variables (Table 1). Substrate was given as the most dominant substrate size in seven categories: 1) Silt, sand and gravel, 2) sand, 3) gravel and boulder, 4) gravel, 5) silt and sand, 6) sand and gravel, and 7) rock and gravel. Width, depth and flow velocity were given as average values of the sampling site. Total phosphorus (TP), nitrate (NO_3_^-^) and alkalinity (meq L^-1^ CaCO_3_) were given as annual mean values. These parameters were selected due to an adequate availability in the present dataset. Missing values for the environmental data were assumed to be missing at random.

**Table 1:** Number of observations per category of the categorical variables (including missing values), and the quantiles (5, 50, 95%), mean and percentage of missing values of the continuous variables.

Substrate (number of observations)							
Silt, sand and gravel	Sand	Gravel and boulder	Gravel	Silt and sand	Sand and gravel	Rock and gravel	*Missing*
662	339	279	255	219	54	34	54
**Continuous variables**							
**Quantiles**	**Alkalinity**	**Velocity**	**Nitrate**	**Total phosphorus**	**Width**	**Depth**	
	**(meq CaCO**_**3**_ **L**^**-1**^**)**	**(m s** ^**-1**^ **)**	**(mg L** ^**-1**^ **)**	**(μg L** ^**-1**^ **)**	**(m)**	**(m)**	
5%	0.4	0.10	0.11	19	2	0.1	
50%	2.6	0.50	2.51	110	8	0.4	
95%	6.6	0.80	17.00	796	82	1.5	
Mean	2.9	0.48	4.25	256	18	0.5	
*Missing (%)*	52	60	17	35	14	28	

### 2.2 Species grouping

For delineating species groups, we created a presence-absence matrix of species and sampling sites. Based on this matrix, we clustered species with Jaccard index and the Wards D criterion. We subsequently created 2 to 62 clusters (from now on referred to as ‘species groups’, indicating the target variable), with the last 62^nd^ cluster representing an individual group for each species. For each of the cluster analyses we assessed how well a Random Forest (RF) model assigned the individual species to a group with the environmental data (predictor variables). RF is a machine-learning method that operates by constructing a multitude of decision trees for predictive modelling [21]. The cluster analysis was performed on all sampling sites, but the RF model was trained on a randomly selected 80% of the combined biotic and environmental data, while 20% were used as single validation fraction. We selected the number of species groups before Cohen’s kappa, calculated on the Out-Of-Bag (OOB) predictions, dropped below a value of 0.3 [22]. Hence, the selection of how many species groups are relevant depended on the ability of the model to discriminate between them. It has to be noted that, contrary to the concept of macrophyte communities [see, for instance, 17] this approach does not allow for classifying species into multiple groups.

A model trained to predict the groups before Cohen’s kappa dropped below 0.3 were further tuned by changing the “nodesize” argument (regulating the tree depth). After the model was fine-tuned, it was again applied on the holdout fraction (20%) to preclude strong overfitting. To see how well the model generalizes, we applied 10-kfold cross-validation with the *rfUtilities* package for R [23] on the full dataset, but only after the prediction on the holdout fraction was found reasonable (~5% deviation from OOB predictions).

Two final models were created based on different approaches to handle missing values of the predictor variables (= imputation) to explore if a less complex approach would be outperformed by a more complex approach. For the first approach (less complex), we used the median of the respective predictor variables to substitute missing values. For the second approach (more complex), values were imputed with the missForest package for R [24]. The number of trees for the missForest package was set at 200 and categorical variables were under sampled on the least occurring group. Under sampling indicates that each generated tree in the RF model has an equal number of observations for each group.

The RF models were created with the randomForest package for R [25]. The performance on the OOB and holdout predictions was assessed based on the accuracy and Cohen’s kappa calculated with the *caret* [26] and *rel* [27] packages for R. The accuracy is the percentage of species that the model has classified correctly as belonging to a specific group. Cohen’s kappa takes the hypothetical probability of chance agreement into account, estimates how far the model is deviating from random guessing and informs on the balance of the predictions. The ranges of Cohen’s kappa can be interpret as follows: < 0 = poor agreement, 0–0.19 = slight agreement, 0.20–0.39 = fair agreement, 0.40–0.59 = moderate agreement, 0.60–0.79 = substantial agreement, and ≥ 0.80 = almost perfect agreement [28]. For each model, the number of random bootstrapped predictors at each node (“mtry”) was set to 1 and the number of trees created was set at 7000. The classes were under sampled based on the number of observations in the minority group. Indicating that each tree in the model was create on a random and equal number of observations from each class. The argument “nodesize” was finally set to 200 to reduce effects of overfitting and improving generalization, regulating the number of species occurrences that end up in the terminal nodes.

### 2.3 Discriminative ranges

The discriminative ranges of the species groups along the gradients of environmental variables were analysed with the fine-tuned RF models. We extracted the mean, maximum and minimum values of the split-points of the continuous variables when they occurred in the root-node of the generated trees. Since the number of trees was set at 7000 and “mtry” at 1 each variable ended up ~1000 times in the root-node, as there were seven predictors. This allowed to quantify the variability of the split-points, where the discrimination between groups was most apparent. The wider the ranges, the more variable the split-points of the RF model occurred in the root-node. The ranges of the split-points were defined as discriminative ranges. The predictions and discriminative ranges of the models were displayed in partial dependency plots (PDPs), created with the *pdp* package for R [29]. This was not possible for the categorical variable ‘substrate type’, and only the category for which a species group is most likely predicted was displayed. The PDPs show the marginal “effect” one predictor has on the voting fraction of the model. All calculations were performed in R [30] and the additional R packages used were *ggplot2* [31] and *cowplot* [32].

## 3. Results

A four-cluster solution represented the highest number of different species groups before Cohen’s kappa dropped below 0.3 (Fig 2). Accuracy and Cohen’s kappa for both approaches to handle missing values were similar (around 50–55% and 0.35–0.40, respectively) (S2 and S3 Tables). Species group 1) comprised mosses such as *Amblystegium fluviatile* and *Fontinalis antipyretica*, species group 2) shorter and pioneer species such as *Callitriche* spp., species group 3) emergent and floating species such as *Sagittaria sagittifolia* and *Lemna* spp., and species group 4) eutraphent species such as *Myriophyllum spicatum* and *Stuckenia pectinata*.

**Fig 3:**
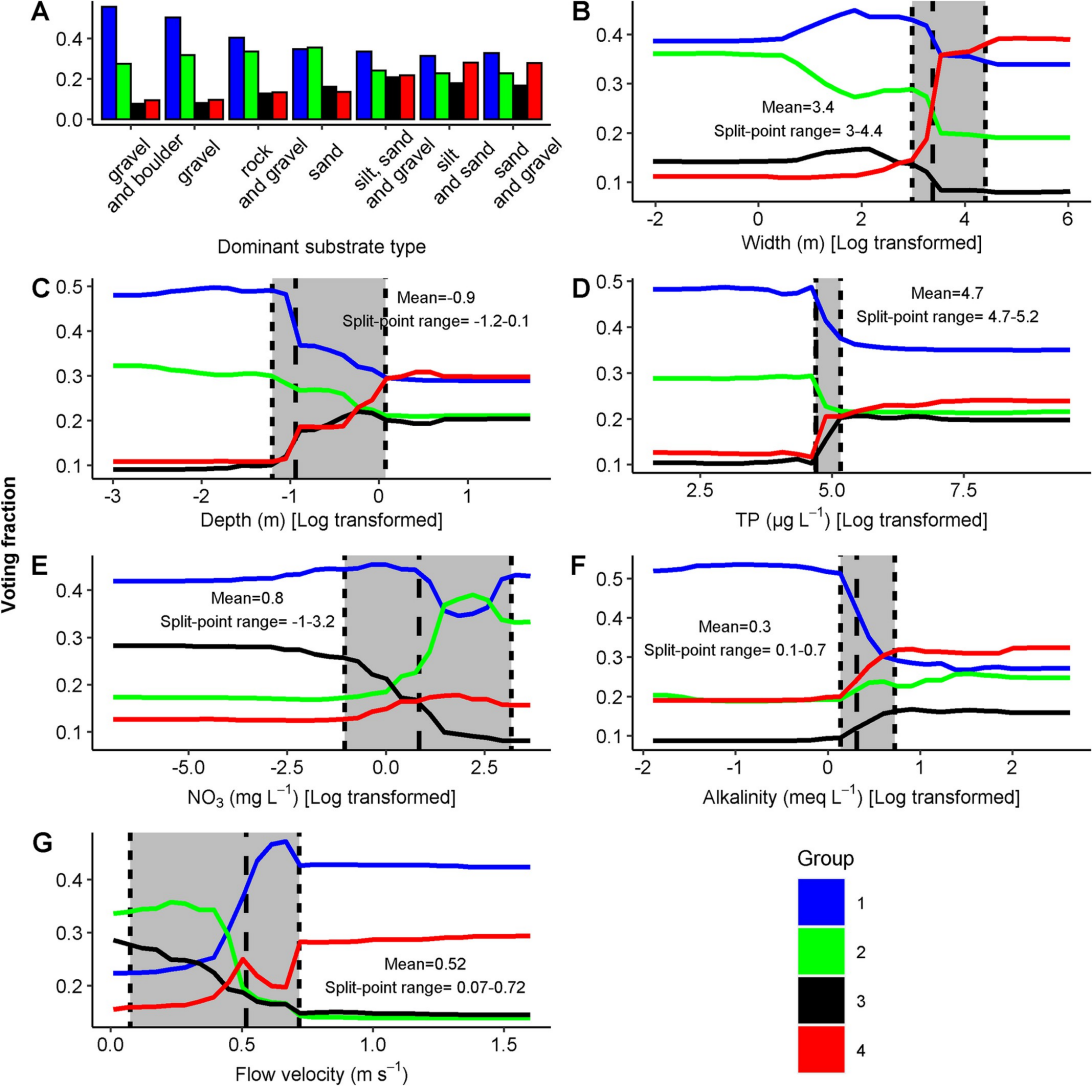
Partial dependency plots of Groups 1–4 displaying predictions of the RF model. A) dominant substrate type, B) width, C) total phosphorus, D) depth, E) alkalinity, F) nitrate and G) flow velocity. Grey coloured areas indicate the split-point range representing the minimum and maximum values extracted from the root-nodes for each generated tree. The two dotted lines indicate the minimum and maximum values and the dashed line indicates the mean. Variables are log-transformed for visualization (natural logarithm).

Species in Group 1 were predicted to occur more likely on coarse substrate (boulder, rocks or gravel) and species in Group 4 were predicted to occur more likely on fine substrate (silt, sand or gravel). Species in Group 2 had an intermediate position, predicted on coarse substrate but less than species in Group 1 and more than species in Group 3 (Fig 3A). Width and depth affected the discrimination as well: Species in Group 4 were predicted to occur more likely in wider (> 20–82 m) and deeper (> 0.3–1.1 m) rivers, while this was the opposite for species in Group 2. Species in Group 3 were predicted to occur more likely in smaller (< 20–82 m) but deeper rivers (> 0.3–1.1 m). Species of Group 1 were predicted to occur more likely in shallow rivers (< 0.3–1.1 m) and showed a unimodal response towards occurrence in medium-sized rivers, although width was less influential (Fig 3B).

The nutrients TP and NO_3_^-^ also played an important role. Species in Group 1 were predicted to occur more likely at TP concentrations lower than 109–164 μg L^-1^ (Fig 3D), while Group 3 and 4 species were predicted to occur more likely at concentrations above 109–164 μg L^-1^. The response to NO_3_^-^ emerged far at the end of the gradient, and Group 1 and 4 showed limited response, whereas Group 3 was discriminated more likely under lower NO_3_^-^ concentrations and Group 2 under concentrations higher than 0.5–22 mg L^-1^, respectively (Fig 3E). Group 2 species were predicted to occur more likely at higher NO_3_^-^ (see also S1 Fig). Group 1 species were predicted to occur more likely at alkalinities lower than 1.1–2.0 meq L^-1^, while predictions of Group 2 were uncertain (Fig 3F). Group 3 and 4 species were predicted to occur more likely at alkalinities above 1.1–2.0 meq L^-1^. For velocity, both Group 1 and 4 species were predicted to occur more likely above 0.07–0.70 m s^-1^, while Group 2 and 3 species were predicted to occur less frequently above 0.07–0.70 m s^-1^ (Fig 3G).

## 4. Discussion

We discriminated four macrophyte groups with a combination of cluster analyses and RF models. These four groups represent those species compositions which occurred most often together at different sampling sites. The accuracy and Cohen’s kappa for both imputation methods for missing values were similar to another study predicting the presence/absence of macrophytes [22]. The overall strong variability of macrophyte occurrence along the gradients does not make the more complex and precise imputation with missForest superior to simply using the median. Furthermore, the fraction of variables missing for particular parameters might be too large for missForest. Hence, there is no clear pattern to “recognize” by missForest that would lead to outperforming the simple imputation using the median.

### 4.1 Macrophyte groups

Group 1 comprises species of shallow mountain brooks with coarse substate, high flow velocities and low alkalinity and TP concentrations. This group represents the so called “moss zone” of headwaters, where mosses often occur under high flow velocities and attach to coarse substrate [33–35]. Therefore, mosses such as *Amblystegium fluviatile*, *Chiloscyphus polyanthos* and *Fontinalis antipyretica* can often be found together as described by [36] [Belgium].

Group 2 comprises species found in shallow streams under low TP concentrations and flow velocity. Most of these species are either small, or have floating leaves and are rooted in the sediment or are fast colonizers (e.g., *Callitriche spp*., *Glyceria*. *fluitans*, *Polygonum amphibium* and *Potamogeton natans*). Other species in this group occur at the banks of lowland brooks (*Nasturtium officinalis* and *Veronica beccabunga*) or are mosses tolerant to eutrophication (e.g., *Amblystegium tenax* [37]). This group shows similarities to groups described by [36,38–41] [Belgium, Italy, Great Britain, France and Denmark].

Group 3 comprises species often occurring in deep and wide rivers under high alkalinity and TP concentrations but low flow velocities. Species include free-floating (*Lemna* spp. and *Spirodela polyrhiza*), common lowland species (e.g., *Sparganium emersum*) and species growing on banks and shallow channel zones (*Berula erecta* and *Scirpus lacustris*). Species with large leaves or free-floating are particularly susceptible to higher flow velocities and can be easily damaged, de-rooted or flushed downstream [13,42–44]. Similarities are present with [38] [Denmark], sharing the observation that *Elodea canadensis*, *Lemna minor* and *Sparganium emersum* occur in similar clusters.

Group 4 comprises species such as *Ceratophyllum demersum*, *Myriophyllum spicatum*, *Potamogeton crispus* and *Stuckenia pectinata* that can tolerate the extremes of the environmental variables investigated in this study. These species are often associated with large eutrophic rivers [37,45], are able to utilize both HCO_3_^-^ and CO_2_ [46], can tolerate high levels of salinity [47], form long shoots [5] and can adapt their physiology [13]. Hence, the groups can be separated based on traits or adaptive strategies fitting with the concurrent environmental variables.

A drawback of our methodology is that the model cannot discriminate multiple species in different groups. For example, Group 3 and 4 cannot both contain *Potamogeton crispus*. A possible workaround could be to weigh (w) the groups (g) or species within the GINI index: GINI = Ʃ(1 - (g_i_*w_i_-…-g_n_*w_n_)^2^). Better discriminating groups get a higher weight compared to poorly discriminating groups. This would allow the algorithm to maximise GINI over higher weighted groups in each tree, which affects the error-rate and location of the split-points.

### 4.2 Discriminatory ranges

The observed ranges of variables in this pan-European dataset fit well with both expert judgement and experiments from literature originating from very different regions. However, the imputation of missing values might change the precision of the results. Nonetheless, the hydromorphological characteristics substrate, width and depth were of importance, with flow velocity to a lesser extent. Substrate was clearly decisive for Group 1 and 4 species. Substrate reflects hydrological regime [15], habitat heterogeneity [48] and nutrient availability [49]. It is a crucial proxy variable reflecting a multitude of environmental conditions changing along the river continuum [50,51]. More stable coarse substrate is representative for higher flow velocities and lower nutrient contents, whereas finer substrate often goes along with lower flow velocities and higher nutrient contents. It can potentially indicate eutrophication processes including the release of nutrients to the surface water [52].

The separation of the groups along the gradients of width and depth resulted in relatively broad discriminatory ranges. For width (~20–80 m) and depth (~0.3–1.0 m), the ranges correspond to the classes of Schaumburg et al. [53], who placed boundaries to distinguish between river types at 40 m width and 0.3 m depth. Riis and Biggs [54] suggested a boundary at 1.0 m and van Geest [55] described and optimum for submerged plants at 0.5–1.0 m depth in lakes. The prediction of Group 4 strongly increases with width, while the predictions of the other groups slightly declined. Wider and deeper rivers are relatively fast flowing and have a high trophic state, thus only a few larger and tolerant species regularly occur, as in Group 4.

The chemical variable TP showed a narrower range compared to width, depth and flow velocity. The discriminative ranges of TP were between 109–112 μg L^-1^, similar to observed changes in macrophyte composition and disappearance in lakes [56,57]and rivers [58,59]. Novak and Chambers [58] suggested management should focus to reduce TP in rivers below ~100–150 μg L^-1^, as the diversity of species declined beyond this point. Presumably, diversity decline is caused by increasing dominance of algae or by competition between species of Groups 3/4 with species of Groups 1/2. Giblin et al. [59] showed that free-floating plants (mainly present in our Group 3) increase in abundance at TP ranges of 43–167 μg L^-1^. However, species related to more nutrient-rich conditions were also included in Group 2 (e.g., *Lemna gibba* n = 28 and *Zannichellia palustris* n = 41). This is probably related to the low number of observations for these species in our dataset, not representing their real distribution. It is yet to be noted that various authors challenged the role of TP as explanatory factor for macrophyte distribution due to absence of a direct known mechanistic link and spurious correlations [60–62].

For nitrate, discriminative ranges were broader (~0.5–20 mg L^-1^), which is in line with [63], who also found broad ranges for species responding positively to nitrate (2.5‐9.7 mg L^-1^). Yet, the range is extremely broad suggesting smooth transition along the gradient instead of “breakpoints” at particular nitrate concentrations. Similar broad ranges have been observed for *Chara hispida* and *C*. *vulgaris*, which show relatively constant growth rates up to 30 mg L^-1^ [64]. Moreover, growth inhibiting effects at relatively low nitrate concentrations may play a role as well. Boedeltje et al. [65] suggested that for species dominantly assimilating ammonium (e.g., *Potamogeton alpinus*), the switch to nitrate as the main source of nitrogen comes at high metabolic costs reducing the growth rate. In our analysis, *P*. *alpinus* occurred in Group 3 that is related to lower nitrate concentrations. In contrast, Group 2 species were predicted to occur more likely under higher NO_3_^-^ concentrations. This could be the result of higher denitrification rates in downstream sections, where anaerobic sediment is more dominant [66]. So these differences between groups might simply reflect the river continuum. Hence, the mechanistic rational of nitrate is not clear in relation to macrophytes or functional groups.

For alkalinity, we found discriminative ranges of ~1.0–2.0 meq L^-1^, which is also in line with the observations of other authors. For example, Butcher [50] termed the concentration range of 0.4–2.0 meq L^-1^ as “slightly calcareous and almost neutral”, below 0.4 meq L^-1^ as non-calcareous and above 2.0 meq L^-1^ as alkaline. Moyle [67] suggested the distinction between soft and hard water lakes between 0.6–1.0 meq L^-1^ (30–50 ppm), Arts et al. [14] between 1.0–2.0 meq L^-1^ and, recently, Lyche Solheim et al. [16] set boundaries for river types at 1.0 meq L^-1^. The mechanistic link is likely the result of carbon concentrating mechanisms [7], but why these particular boundaries were observed remains unclear.

Flow velocity also displayed recognizable patterns. We observed a discriminatory range of ~0.05–0.70 m s^-1^, which is in line with the observation of other authors [11,15,34]. Biggs [15] suggested that assemblages change from vascular plants to mosses around 0.3–0.7 m s^-1^ and French and Chambers [34] showed that after 0.4–0.6 m s^-1^ vascular plants are often absent. Chambers et al. [68] noted that velocities over a range of 0.2–0.7 m s^-1^ coincided with a decrease in plant biomass. The figures given by Kemp et al. [11] showed changes around 0.5 m s^-1^ and it was observed that above 0.5 m s^-1^ submerged fine leaved macrophytes dominated. Also Smidt et al. [69] noted that medium to fast flowing was between 0.35–0.70 m s^-1^. Moreover, Kemp et al. [11] observed that emergent macrophytes preferred up to 0.05 m s^-1^, coinciding with our Group 3. We showed that the voting fraction starts to increase at ~0.3 m s^-1^ (see Fig 3). *C*. *demersum* was also placed in Group 4, but it is not expected under higher flow velocities. This species likely grows closer to the banks sheltered from higher flow velocities. This confirms that the measurements of the environmental variables are averages of presumably heterogeneous conditions within a river stretch.

The discriminatory ranges are not to be mistaken with management thresholds representing points along a gradient at which a desired status is likely to be achieved (e.g., distinct water or habitat quality targets) [70]. They also neither suggest a clear discrimination for each unique gradient nor group predictions independent from other (measured and unmeasured) environmental variables. Furthermore, the discriminative ranges do not indicate that a species is restricted to higher/lower parts of the environmental gradient. These ranges rather denote that the discrimination of the model between the groups was most noticeably at this range along the gradient.

Causation and independence can in most cases not be derived based on data obtained by biological monitoring programmes. Our analysis is thus associative and exploratory rather than causative. The dataset used is not suited to study nuanced cause-effect relations, because environmental gradients are correlated, and confounding factors are not controlled for. Despite these limitations, the dataset’s coverage of large spatial and environmental gradients allows for detecting patterns that conform to findings of other studies. This is beneficial for defining valid macrophyte groups and the corresponding environmental conditions, particularly in the light of ecosystem management and recovery at the European scale [16,17]. Our results and their discussion also provide evidence to build informed priors for Bayesian inference (e.g. Bayesian threshold/changepoint analysis) or to substantiate “breakpoints” in diagnostic networks [71]. Yet, the most interesting but open questions are: Which mechanisms are the result of these observed discriminatory ranges? Why do they exactly occur at these locations, and can they be approximated with theoretical models?

As demonstrated above, the macrophyte groups resulting from our analysis show strong similarities to group descriptions in various national macrophyte studies. Our discriminatory ranges for the relevant environmental factors provide quantitative evidence instead of qualitative accounts derived from small-scale investigations or expert judgment. The discriminatory ranges fit well with these accounts, suggesting that they are generalizable across different spatial scales. Our study may initiate further research on the multiple factors determining macrophyte occurrence beyond the usual focus on the trophic state. Our study may initiate further research on broader concepts for macrophyte occurrence beyond the usual focus on trophic state. An improving capacity to quantify the environmental conditions that determine the presence of riverine macrophytes will ultimately benefit biological prediction, facilitating enhanced conservation and restoration effectiveness.

## Supporting information

S1 Fig Species occurrence along the NO₃¯ gradient (sorted by decreasing 25th percentile values). (TIF)

S1 Table Table of respective providers for the used dataset. (DOCX)

S2 Table Performance measures of the model where missing values were replaced by the median and the model was assessed on the datasets excluding samples with missing values. (DOCX)

S3 Table Performance measures of the model where values were imputed with the missForest package at the location where no values were available. (DOCX)
