# Supplementary figures and images for: Environmental ranges discriminating between macrophytes groups in European rivers
Source: PLoS One. 2022 Jun 14;17(6):e0269744. doi: 10.1371/journal.pone.0269744 (PMC9197031; doi:10.1371/journal.pone.0269744)

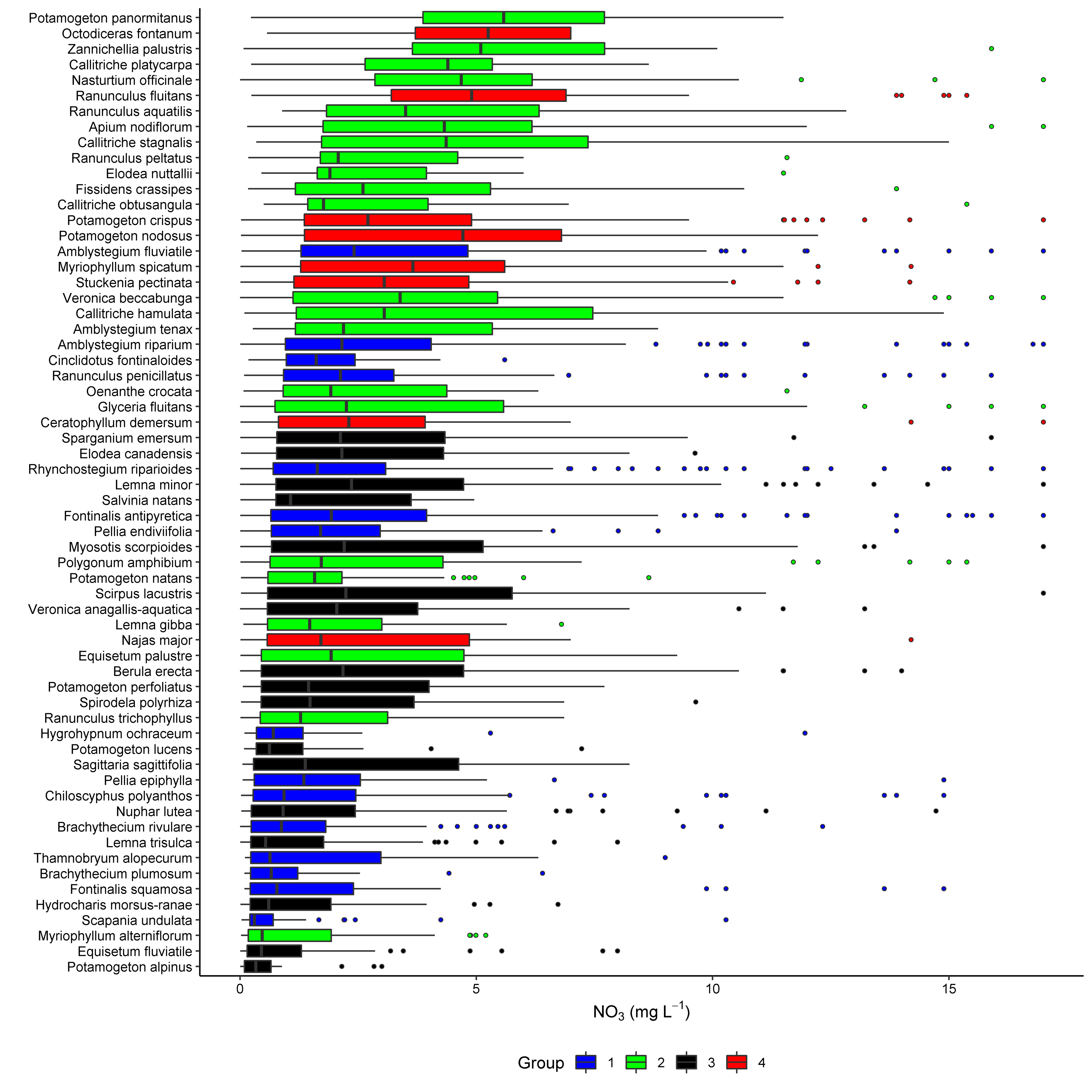

Supplement: S1 Fig — (TIF) [file pone.0269744.s001.tif]
