# Supplementary material for: Environmental ranges discriminating between macrophytes groups in European rivers
Source: PLoS One. 2022 Jun 14;17(6):e0269744. doi: 10.1371/journal.pone.0269744 (PMC9197031; doi:10.1371/journal.pone.0269744)
Supplement: S1 Table — (DOCX) [file pone.0269744.s002.docx]

S1 Table: Table of respective providers for the used dataset.

| **Name** | **Country (abbreviated)** | **Name** | **Country (abbreviated)** |
| --- | --- | --- | --- |
| Agnieszka Kolada | PL | Jens Kroker | DE |
| Alain Dohet | LU | Jochen Schaumburg | DE |
| An Leyssen | BE (Flanders) | Jürgen Böhmer | DE |
| Andreas Hoppe | DE | Kai Piirsoo | EE |
| Anik Schneiders | BE (Flanders) | Karin Pall | AT |
| Annette Baattrup-Pedersen | DK | Klaus van de Weyer | DE |
| Annette Baattrup-Pedersen | UK (GB) | Kris van Looy | BE (Flanders) |
| Annette Baattrup-Pedersen | DE | Krzysztof Szoszkiewicz | PL |
| Antun Alegro | HR | Lenka Tajmrova | CZ |
| Béla Csanyi | HU | Libuse Opatrilova | CZ |
| Brigitte Schmidt | AT | Livia Tothova | SK |
| Chris Burns | UK (NI) | Lukács András | HU |
| Christian Chauvin | FR | Maria Minicardi | IT |
| Christine Schranz | DE | Martin Mcgarrigle | IE |
| Christophe Laplace | FR | Matus Haviar | SK |
| Daniel Galoux | BE (Wallonia) | Nigel Willby | UK (GB) |
| Doris Stelzer | DE | Nora Welschbillig | LU |
| Eva Bellack | DE | Normunds Kadikis | LV |
| Gana Gecheva | BG | Peter Balazi | SK |
| Gorazd Urbanic | SI | Peter Rolauffs | DE |
| Henar Fraile | ES | Roelf Pot | NL |
| Hugo Coops | NL | Sebastian Birk | STAR (project) |
| Isabel Pardo | ES | Serban Iliescu | RO |
|  |  | Ulrich Schmieds | DE |
|  |  | Zofija Sinkevičienė | LT |
