# Supplementary material for: Environmental ranges discriminating between macrophytes groups in European rivers
Source: PLoS One. 2022 Jun 14;17(6):e0269744. doi: 10.1371/journal.pone.0269744 (PMC9197031; doi:10.1371/journal.pone.0269744)
Supplement: S2 Table — (DOCX) [file pone.0269744.s003.docx]

S2 Table: Performance measures of the model where missing values were replaced by the median and the model was assessed on the datasets excluding samples with missing values.

|  | Out-Of-Bag performance | | | | Holdout performance | | | | 10 kfold-cross validation | | | |
| --- | --- | --- | --- | --- | --- | --- | --- | --- | --- | --- | --- | --- |
| Accuracy | 50% (LCI=49%; HCI%=52%) | | | | 52% (LCI=50%; HCI=55%) | | | | 55% | | | |
| Cohen's kappa | 0.33 (LCI=0.32; HCI=0.35) | | | | 0.37 (LCI=0.33; HCI=0.40) | | | | 0.38 | | | |
| n | 6472 | | | | 1618 | | | | 8090 | | | |
| Groups | 1 | 2 | 3 | 4 | 1 | 2 | 3 | 4 | 1 | 2 | 3 | 4 |
| Prevalence | 0.29 | 0.24 | 0.32 | 0.15 | 0.29 | 0.23 | 0.33 | 0.15 | 0.29 | 0.24 | 0.32 | 0.15 |
