# Supplementary material for: Environmental ranges discriminating between macrophytes groups in European rivers
Source: PLoS One. 2022 Jun 14;17(6):e0269744. doi: 10.1371/journal.pone.0269744 (PMC9197031; doi:10.1371/journal.pone.0269744)
Supplement: S3 Table — (DOCX) [file pone.0269744.s004.docx]

S3 Table: Performance measures of the model where values were imputed with the missForest package at the location where no values were available.

|  | Out-Of-Bag performance | | | | Holdout performance | | | | 10 kfold-cross validation | | | |
| --- | --- | --- | --- | --- | --- | --- | --- | --- | --- | --- | --- | --- |
| Accuracy | 52% (LCI=51%; HCI%=53%) | | | | 51% (LCI=49%; HCI=54%) | | | | 54% | | | |
| Cohen's kappa | 0.35 (LCI=0.34; HCI=0.37) | | | | 0.34 (LCI=0.31; HCI=0.37) | | | | 0.37 | | | |
| N | 6472 | | | | 1618 | | | | 8090 | | | |
| Groups | 1 | 2 | 3 | 4 | 1 | 2 | 3 | 4 | 1 | 2 | 3 | 4 |
| Prevalence | 0.29 | 0.24 | 0.32 | 0.15 | 0.29 | 0.23 | 0.33 | 0.15 | 0.29 | 0.24 | 0.32 | 0.15 |
